# Costs and cost‐effectiveness of a collaborative data‐to‐care intervention for HIV treatment and care in the United States

**DOI:** 10.1002/jia2.26040

**Published:** 2023-01-22

**Authors:** Ram K. Shrestha, Robyn Neblett Fanfair, Liisa M. Randall, Crystal Lucas, Lisa Nichols, Nasima Camp, Kathleen A. Brady, Heidi Jenkins, Frederick L. Altice, Alfred DeMaria, Merceditas Villanueva, Paul J. Weidle

**Affiliations:** ^1^ Division of HIV Prevention National Center for HIV Viral Hepatitis STD and TB Prevention Centers for Disease Control and Prevention Atlanta Georgia USA; ^2^ Massachusetts Department of Public Health Boston Massachusetts USA; ^3^ Philadelphia Department of Public Health Philadelphia Pennsylvania USA; ^4^ Yale University School of Medicine New Haven Connecticut USA; ^5^ Connecticut Department of Public Health Hartford Connecticut USA

**Keywords:** HIV surveillance, data‐to‐care, re‐engagement, randomized controlled trial, economic evaluation, cost‐effectiveness

## Abstract

**Introduction:**

Data‐to‐care programmes utilize surveillance data to identify persons who are out of HIV care, re‐engage them in care and improve HIV care outcomes. We assess the costs and cost‐effectiveness of re‐engagement in an HIV care intervention in the United States.

**Methods:**

The Cooperative Re‐engagement Control Trial (CoRECT) employed a data‐to‐care collaborative model between health departments and HIV care providers, August 2016–July 2018. The health departments in Connecticut (CT), Massachusetts (MA) and Philadelphia (PHL) collaborated with HIV clinics to identify newly out‐of‐care patients and randomize them to receive usual linkage and engagement in care services (standard‐of‐care control arm) or health department‐initiated active re‐engagement services (intervention arm). We used a microcosting approach to identify the activities and resources involved in the CoRECT intervention, separate from the standard‐of‐care, and quantified the costs. The cost data were collected at the start‐up and recurrent phases of the trial to incorporate potential variation in the intervention costs. The costs were estimated from the healthcare provider perspective.

**Results:**

The CoRECT trial in CT, MA and PHL randomly assigned on average 327, 316 and 305 participants per year either to the intervention arm (*n* = 166, 159 and 155) or the standard‐of‐care arm (*n* = 161, 157 and 150), respectively. Of those randomized, the number of participants re‐engaged in care within 90 days in the intervention and standard‐of‐care arms was 85 and 70 in CT, 84 and 70 in MA, and 98 and 67 in PHL. The additional number of participants re‐engaged in care in the intervention arm compared with those in the standard‐of‐care arm was 15 (CT), 14 (MA) and 31 (PHL). We estimated the annual total cost of the CoRECT intervention at $490,040 in CT, $473,297 in MA and $439,237 in PHL. The average cost per participant enrolled was $2952, $2977 and $2834 and the average cost per participant re‐engaged in care was $5765, $5634 and $4482. We estimated an incremental cost per participant re‐engaged in care at $32,669 (CT), $33,807 (MA) and $14,169 (PHL).

**Conclusions:**

The costs of the CoRECT intervention that identified newly out‐of‐care patients and re‐engaged them in HIV care are comparable with other similar interventions, suggesting a potential for its cost‐effectiveness in the US context.

## 1 INTRODUCTION

There are an estimated 1.2 million people with HIV (PWH) in the United States. The clinical practice guidelines for HIV recommend initiating antiretroviral therapy for everyone at diagnosis [1]. Among persons with HIV, 86% are diagnosed and 65% received HIV care, 50% are engaged in care, and 56% achieved viral suppression [2]. Sustained viral suppression is a critical step in HIV treatment, and leads to improved health, quality of life and life expectancy. Further, the persons achieving viral suppression and maintaining an undetectable viral load have effectively no risk of transmitting HIV to their sexual partners [3]. Despite the individual and public health benefits of HIV treatment, engaging and retaining PWH continuously in HIV care has been challenging. The federal government launched a new initiative, Ending the HIV Epidemic in the United States (EHE) in 2019, with the aim to substantially increase HIV prevention efforts and reduce the number of new HIV transmissions by 75% in 2025, and by 90% in 2030 [4, 5].

Data to care (D2C) is a public health strategy where routine surveillance data are used to identify PWH who are out of care, engage them in HIV medical care and achieve viral suppression [6, 7, 8, 9]. A collaborative D2C model may be initiated by health departments with primary healthcare providers, or *vice versa*, by using HIV case surveillance data and other data sources [8, 10]. The literature shows some success in implementing D2C and improving HIV care outcomes [11, 12, 13, 14, 15].

The Centers for Disease Control and Prevention (CDC) funded the Cooperative Re‐Engagement Control Trial (CoRECT) to implement a collaborative D2C model and evaluate effectiveness and costs [16]. CoRECT was a randomized controlled multi‐site trial that utilized D2C to identify persons with HIV who were newly out of care and re‐engage them in HIV medical care, retain them in care and achieve viral suppression. We assess the costs and cost‐effectiveness of the CoRECT intervention in terms of cost per person re‐engaged in care, which included identifying persons who were newly out of HIV care and re‐engaging them in care, using primary data on intervention effectiveness and costs obtained from the trial.

## 2 METHODS

### 2.1 Study design

The CoRECT trial used routine HIV surveillance data to identify newly out‐of‐care patients and re‐engage them in HIV care. The process included screening eligible newly out‐of‐care participants for enrolment in the study, and assigning participants to one of two study arms, where participants were re‐engaged in medical care through either standard practice or active public health field service assistance [16]. Screening participants for eligibility involved several steps, including the generation of out‐of‐care lists using HIV laboratory data reported to health department surveillance programmes, reconciliation of the surveillance out‐of‐care list with a list generated by the HIV care clinics using missed appointment data, eligibility determination—excluding those deceased, incarcerated or moved out of jurisdictions, and case conferences with clinics to generate a final list of participants for randomization. Newly out‐of‐care was defined as a person who had received HIV medical care at the clinic for 12 months and then disengaged from care, that is no clinic visit or CD4 count or HIV viral load recorded for more than 6 months [16, 17].

All participants deemed out‐of‐care were randomized to either the standard‐of‐care (SOC) control arm, where they received routine clinic re‐engagement in care services involving communication via telephone calls, letters or email by clinic staff, or the CoRECT intervention arm, where they received an active public health field services re‐engagement assistance, in addition to the SOC services. The active field services for re‐engagement were provided by the health department field epidemiologists or disease intervention specialists (DIS). The DIS/field epidemiologist re‐engagement activities included reviewing participant records, outreach to locate and contact participants, providing re‐engagement assistance, such as transportation assistance, same‐day appointments, appointment reminders and follow‐up with the engagement assistance. Participants were enrolled in the trial on a rolling basis; recruitment began in August 2016 and ended in July 2018. The study was approved by each health department's Institutional Review Board.

The primary outcomes of the trial included: (1) re‐engagement in care–defined as CD4 count or viral load reported to surveillance programmes within 90 days of randomization; (2) retention in care–defined as at least two CD4 counts or viral loads reported ≥3 months apart within 12 months post‐randomization; and (3) viral load suppression–defined as one viral load (<200 copies/ml) reported by 12 months of randomization. The trial design, intervention and study outcomes are described in detail elsewhere [16]. Because the CoRECT trial did not demonstrate the effectiveness of retention in care in two of the three jurisdictions and viral suppression in any of the three [16], our costs and cost‐effectiveness analysis focuses only on the first outcome—re‐engagement in care.

### 2.2 Study setting and site description

The CoRECT study was implemented by the Connecticut Department of Public Health (CT); the Massachusetts Department of Public Health (MA); and the Philadelphia, PA (PHL), Department of Public Health. The health departments collaborated with multiple clinics and facilities providing HIV care in their jurisdictions to implement the trial. The organizational and operational details of the sites are provided elsewhere [16, 17].

### 2.3 Cost data collection and analysis

We used microcosting methods to estimate the economic cost of the CoRECT intervention, which included identifying persons who were newly out of HIV care and re‐engaging them in care through the active public health field service re‐engagement assistance. Microcosting involves detailed inventory and enumeration of all variable and fixed costs attributable to the intervention; the “variable cost” varies with the number of participants served, whereas the “fixed cost” remains the same regardless of the number of participants [18, 19, 20, 21, 22, 23]. First, we identified the intervention activities and resources involved during all phases of the CoRECT intervention. Second, we developed cost data collection tools in collaboration with the principal investigators and field operation staff to collect costs specifically attributable to the CoRECT interventon, and pilot‐tested the cost form for completeness. Third, the health department and collaborating clinics used the final form to report the intervention cost data within 3 months of the trial initiation (i.e. out‐of‐care participants recruitment) in August 2016 and at 6 and 12 months after the trial initiation. We defined start‐up costs as those incurred during the first 3 months after the trial initiation and recurring costs as those measured at 6 and 12 months after initiation [17].

At each data collection cycle, the data reflected resources used and costs incurred in the intervention over 30 days, and the costs were multiplied by the cycle length in months to estimate the total costs. The cost form included reporting for several cost categories, including pre‐implementation training for active public health intervention, identification of out‐of‐care participants, DIS/field epidemiologist activities in re‐engaging participants in care, programme administration, office supplies, durable equipment, facility space and utilities. The cost data were reviewed and discussed during project update calls for completeness and consistency. All CoRECT intervention costs, regardless of the source of support, were included in the analysis; the trial development prior to out‐of‐care participant recruitment, research and evaluation costs were excluded.

### 2.4 Cost‐effectiveness analysis

We estimated the annual CoRECT intervention cost separately for each project site, then calculated an average cost per participant who received the CoRECT intervention and per participant re‐engaged in care. We estimated the incremental cost per participant re‐engaged in care; representing the incremental cost‐effectiveness ratio (ICER) by dividing the total CoRECT intervention costs by the number of additional participants re‐engaged in care in the CoRECT intervention arm compared with those in the SOC arm. The intervention costs were considered to be incremental to the SOC costs; we did not collect SOC costs as these services were available to all participants regardless of the study arm. The analysis was conducted from the healthcare provider perspective in that the state and local health departments collaborated with medical clinics to deliver the CoRECT intervention. The costs are reported in 2018 U.S. dollars.

We conducted a sensitivity analysis to evaluate potential reductions in out‐of‐care patient identification costs and fixed costs of the CoRECT intervention if the programme was scaled up in other healthcare settings or jurisdictions. Further, the clinics could potentially streamline re‐engagement activities as the health departments would support many of the re‐engagement efforts, including field service re‐engagement assistance to re‐engage out‐of‐care patients. We conducted a sensitivity analysis to evaluate the impact of potential task shifting on cost‐effectiveness by reducing the intervention cost of the clinics.

## 3 RESULTS

The CoRECT trial in CT, MA and PHL randomly assigned on average 327, 316 and 305 participants per year either to the intervention arm (*n* = 166, 159 and 155) or the SOC arm (*n* = 161, 157 and 150), respectively (Table 1). Of those randomized, the number of participants re‐engaged in care in the intervention and SOC arms was 85 and 70 in CT, 84 and 70 in MA, and 98 and 67 in PHL. The additional number of participants re‐engaged in care in the intervention arm compared with those in the SOC arm was 15, 14 and 31 in CT, MA and PHL, respectively.

**Table 1:** Annual programme costs and health outcomes of the CoRECT intervention

	Connecticut		Massachusetts		Philadelphia	
	Intervention	Standard‐of‐care	Intervention	Standard‐of‐care	Intervention	Standard‐of‐care
Outcome						
Participants enrolled	166	161	159	157	155	150
Participants re‐engaged care^a^	85	70	84	70	98	67
Incremental outcome						
Participants re‐engaged in care	15	–	14	–	31	–
Programme cost						
Total intervention cost ($)	490,040	–	473,297	–	439,237	–
Health department cost ($)	276,179	–	184,396	–	368,352	–
Clinics cost ($)	213,862	–	288,901	–	70,885	–
Labour cost (% of total)	0.95	–	0.98	–	0.95	–
Variable cost (% of total)	0.46	–	0.58	–	0.61	–
Average cost ($)						
Cost per participant enrolled in the intervention	2952	–	2977	–	2834	–
Cost per participant re‐engaged in the intervention	5765	–	5634	–	4482	–
Incremental cost‐effectiveness ($)						
Cost per participant re‐engaged in care	32,669	–	33,807	–	14,169	–

Note: –, is not applicable. Costs are reported in 2018 U.S. dollars.

Abbreviation: CoRECT, Cooperative Re‐engagement Control Trial.

^a^ Number of participants re‐engaged in care in standard‐of‐care (SOC) arm was adjusted, assuming the SOC arm had the same number of participants enrolled as in the intervention arm. For example, in Connecticut, (68/161)x166 = 70, where 68 was an unadjusted number of participants re‐engaged in care.

We estimated the annual total CoRECT intervention cost at $490,040 in CT, $473,297 in MA and $439,237 in PHL (Table 1). Of the total costs, 56%, 39% and 84% were attributable to health departments and 44%, 61% and 16% were attributable to clinics in CT, MA and PHL, respectively. In the CoRECT intervention arm, the average cost per participant enrolled was $2952, $2977 and $2834, and the average cost per participant re‐engaged in care was $5765, $5634 and $4482 in CT, MA and PHL. We estimated an ICER, representing incremental cost per participant re‐engaged in care, at $32,669, $33,807 and $14,169, in CT, MA and PHL, respectively.

The majority of the programme costs were attributable to labour costs across all sites, ranging from 95% to 98% of the total intervention costs (Table 1). We found a similar proportion of labour cost allocation in the health departments across sites (Table 2). However, the total labour hours spent on the intervention varied across sites. In health departments, the labour hours ranged from 2882 hours in MA to 10,340 hours in PHL. The two activities that had the most labour hours spent were general administration (1200 hours) and data management (924 hours) in CT, DIS/field epidemiologists to locate and contact participants (405 hours) and documentation of engagement activities into the databases (281 hours) in MA, and project supervision (1594 hours) and documentation of engagement assistance into the databases (1335 hours) in PHL. All clinic costs were for labour (Table 3). The total labour hours spent in clinics ranged from 1871 hours in PHL to 5816 hours in MA. The two activities that had the most labour hours spent were contacting out‐of‐care participants (1584 hours) and data management (729 hours) in CT, contacting out‐of‐care participant (1307 hours) and generate the out‐of‐care line list (876 hours) in MA, and match with clinic list (546 hours) and case conference (382 hours) in PHL.

**Table 2:** Annual variable and fixed costs of the CoRECT intervention in health departments

	Connecticut		Massachusetts		Philadelphia	
	Labour (hour)	Cost ($)	Labour (hour)	Cost ($)	Labour (hour)	Cost ($)
Variable cost: labour						
Out‐of‐care patient identification						
Generate surveillance line list	26	1930	202	9682	113	3497
Match with clinic list	26	1930	270	12,939	–	–
Communicate with clinic‐data transmission‐initial	17	1249	–	–	65	1948
Health department preliminary investigation	–	–	180	7538	258	8289
Case conference	24	962	266	12,932	311	12,316
Communicate with clinic‐data transmission‐final	13	965	–	–	72	1890
Data entry of final list	24	1817	131	6190	416	14,094
DIS activities						
Records review	117	7583	181	11,916	463	14,667
Outreach to locate and contact out‐of‐care patients	539	34,288	405	19,428	920	28,537
Out‐of‐care interview and barriers to care survey	141	9346	155	7446	993	30,786
Engagement assistance	201	12,561	57	2703	727	22,533
Follow‐up with clinics	127	8533	50	2418	654	20,294
Follow‐up engagement assistance	125	7677	28	1331	366	11,349
Documentation of engagement assistance in the database	230	15,621	281	13,535	1335	41,382
Variable cost: non‐labour						
Office supplies	–	5067	–	205	–	2421
Fixed cost: labour						
Pre‐implementation training	72	4262	36	1782	36	1352
Project‐related meetings	216	18,499	278	27,407	1249	48,026
Data management	924	59,556	57	3149	259	5731
Quality assurance	240	10,686	57	3038	291	10,851
General administration	1200	45,759	95	11,319	218	8296
Project supervision	144	10,795	156	20,247	1594	61,325
Fixed cost: non‐labour						
Electronic equipment	–	4160	–	167	–	921
Office space and utilities	–	12,932^a^	–	9023	–	17,847^a^
Total cost	4403	276,179	2882	184,396	10,340	368,352

Note: –, is not applicable or data not reported.

Abbreviations: CoRECT, Cooperative Re‐engagement Control Trial; DIS, disease intervention specialist.

^a^ Office space and utility costs were calculated as 5.2% of labour costs, based on MA data.

**Table 3:** Annual variable and fixed costs of the CoRECT intervention in HIV care clinics

	Connecticut (*n* = 23)		Massachusetts (*n* = 9)		Philadelphia (*n* = 8)	
	Labour (hour)	Cost ($)	Labour (hour)	Cost ($)	Labour (hour)	Cost ($)
*Variable cost*						
Out‐of‐care patient identification						
Generate out‐of‐care patient list	451	22,174	876	44,660	58	2535
Match with clinic list	523	25,168	–	–	546	19,092
Communicate with health department for data transmission	77	4273	42	2167	37	1355
Case conference	118	6067	163	7370	382	16,292
Patients re‐engagement						
Contacting out‐of‐care patients	1584	54,902	1307	67,506	182	7017
Follow‐up with health department	120	5332	817	41,941	265	8843
Other: addressing patients’ concerns	–	–	–	383	11	353
*Fixed cost: labour*						
Pre‐implementation training and travel	184	13,213	761	32,093	90	3704
Project‐related meetings	230	12,265	734	42,653	48	2238
Data management	729	42,659	649	24,268	25	674
Quality assurance	426	20,342	88	3655	104	3296
General administration	67	3888	169	7758	34	1121
Project supervision	65	3579	210	13,999	29	1184
Other: care team coordination for transition	–	–	–	450	60	3181
Total cost	4574	213,862	5816	288,901	1871	70,885

Note: No. of clinics varied in data reporting cycles, hence, the average cost per clinic was calculated and the average cost was multiplied by no. of clinics (*n*) to estimate the total cost. – is not applicable or data not reported.

Abbreviation: CoRECT, Cooperative Re‐engagement Control Trial.

In all sites, nearly half of the CoRECT intervention costs were attributable to variable costs, ranging from 46% to 61%, and the rest were fixed costs (Table 1). The proportion of variable costs ranged from 40% to 59% in the health departments (calculated from Table 2) and from 55% to 78% in clinics (calculated from Table 3). The CoRECT intervention costs were higher during start‐up than in the recurrent phase in CT and PHL. In the MA health department, the average monthly CoRECT intervention cost was $15,908 during the recurrent phase compared to $13,295 (16% lower) at start‐up (Table S1). Compared to the costs at the recurrent phase, the health department start‐up cost was 11% higher in CT and 6% higher in PHL. In clinics, the start‐up costs were higher in all sites, ranging from 26% to 54% (Table S2).

**Figure 1:**
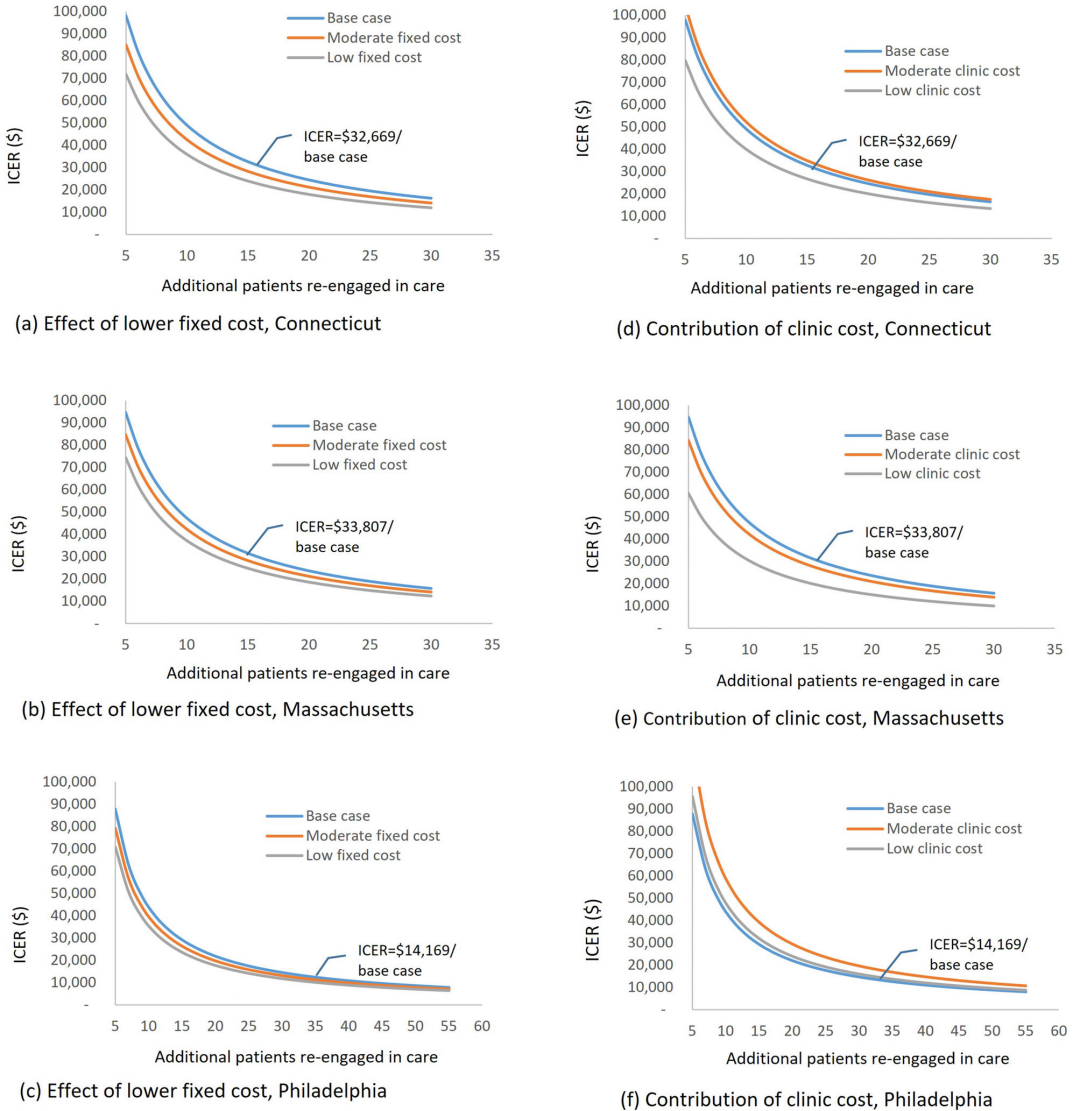
Sensitivity of the intervention cost and cost‐effectiveness of the Cooperative Re‐engagement Control Trial (CoRECT) intervention.Abbreviation: ICER, incremental cost‐effectiveness ratio.

The sensitivity analysis showed that the ICER could be lower and the CoRECT intervention could be more cost‐effective with an increased number of participants re‐engaged in care (Figure 1a–c, base case; Table S3). When we reduced the out‐of‐care patient identification costs by as much as 50% from the base case, the ICER reduced to $31,560, $31,959 and $13,513 in CT, MA and PHA, respectively (Table S3). If we assumed a moderate or low fixed cost (25% or 50% reduction from the base case) and no change in the number of participants re‐engaged in care from the base case, the ICERs were $28,293 and $23,916 in CT, $30,218 and $26,628 in MA, and $12,800 and $11,431 in PHL, respectively. Further, when we assumed that the CoRECT intervention costs in all clinics were 50% of the total cost—moderate clinic cost scenario to account for potential task shifting from clinics to health departments—we estimated ICER at $34,747, $30,075 and $18,967 in CT, MA and PHL (Figure 1d–f and Table S3). Similarly, when we assumed that the clinics’ costs were 25% of the total cost (low clinic cost scenario), the ICER was $26,579, $21,623 and $15,425 in CT, MA and PHL.

## 4 DISCUSSION

The CoRECT study showed a significant improvement in re‐engagement in HIV care at 90 days among PWH newly out‐of‐care who received the CoRECT intervention compared with those receiving SOC. We estimated the costs and cost‐effectiveness of the CoRECT intervention that employed a data‐to‐care collaborative model between health departments and HIV care providers to identify persons who were newly out of HIV care and re‐engage them in care. The health departments contributed from 39% to 84% of the total cost, and the rest was contributed by the clinics. The average cost per CoRECT intervention participant was similar across three sites, ranging from $2834 to $2977. The incremental cost per participant re‐engaged in care was estimated at $33,669 in CT, $33,807 in MA and $14,169 in PHL.

D2C is a public health approach to identify persons with HIV who are out‐of‐care, re‐engage them in HIV medical care and achieve viral suppression [6, 7, 8]. Because of the scope and intensity of the efforts involved in active re‐engagement processes led by DIS/field epidemiologists as implemented, the CoRECT intervention can be costly. The active public health field service re‐engagement assistance provided by the DIS or field epidemiologists involves multiple steps, including assessing barriers to care, motivational interviewing and facilitating appointments to the clinic.

The literature on costs and cost‐effectiveness analysis of other HIV interventions comparable to CoRECT is limited. Maulsby et al. conducted a cost‐effectiveness analysis of the programmes that aimed at finding, linking and retaining hard‐to‐reach persons with HIV infection to medical care [24]. The authors showed that the annual programme costs varied from $339,155 in Montgomery, AL to $1,162,468 in New York City, NY, and the cost per participant varied from $1053 to $3169 (all in 2013 US$), costs which are comparable to our estimate of the average cost per CoRECT intervention participant. The programme was implemented in seven sites, and it was cost‐saving in two sites and cost‐effective in five sites. Another study evaluated the cost‐effectiveness of health information technology supports for patient engagement in HIV care, including the use of HIV surveillance data to identify out‐of‐care patients, the use of electronic laboratory ordering and prescribing, and the development of a patient portal [25]. The study including six programme sites and programme costs varied from $287,862 to $998,201 (2018 US$) over a 3‐year period. The authors showed that the intervention was cost‐saving in three sites and not cost‐effective in two sites.

Our analysis showed that the CoRECT intervention cost was primarily attributable to labour costs, which exceeded 95% of the total cost across all sites, results that are consistent with the literature [25, 26, 27]. A recent microcosting study that evaluated programmes supporting patient engagement in HIV care in six sites showed that labour cost ranged from 73% to 91% of the total programme costs [25]. In our analysis, the clinics reported only labour costs—as the intervention was a small part of the clinics’ overall operation—hence potentially contributing to a slightly higher proportion of labour costs.

Identification of persons who are out of HIV care is a critical step in the CoRECT intervention. In the CoRECT trial, the newly out‐of‐care patients were identified using HIV case surveillance data before they were randomized to receive either SOC or active re‐engagement services. Although most of the data were processed electronically, the case conferences between health departments and clinics to generate the final participants list for randomization may have involved extra cost potentially attributable to those assigned to the SOC arm. We conducted a sensitivity analysis to assess the results with lower out‐of‐care patient identification costs.

Although the total CoRECT intervention costs were similar in three sites ($439,000–$490,000), cost allocation between the health department and clinics varied substantially. This suggests that each site differed in tailoring the intervention to fit the local context, including HIV care coordination and cost‐sharing between the health department and clinics. Further, the variability in costs across sites could be attributable to multiple factors, including differences in wages and fringe benefits, D2C infrastructure and experiences, data management systems and execution of different re‐engagement in care strategies [17].

We collected cost data at start‐up, 6 and 12 months into the trial to account for potential variation in costs at different phases of the CoRECT intervention. The results showed that the monthly average cost at start‐up was generally higher than the cost during recurrent phases, ranging from 6% to 10% in health departments (except MA health department, –20%, as some of the D2C infrastructure was already established) and 21–35% in clinics. However, the costs reported at 6 and 12 months were virtually the same when the sites reported costs in both cycles. Future cost analysis may focus on collecting costs data at start‐up and one of the 6‐ or 12‐month cycles to minimize the data collection burden.

Our analyses have some limitations. First, only the MA health department reported the cost of office space and utilities ($9023; 5.2% of labour costs, Table 2). We assumed the office space and utility costs of CT and PHL at 5.2% of the health department labour costs, consistent with MA. Space and utility costs have differed in other HIV prevention interventions [19, 27]. The cost analysis of the retention in care intervention for patients with HIV (CDC/HRSA trial) showed that the office space and utility costs were 6% of labour (5% of the total) cost [19].

Second, in CT, cost data were collected from a representative sample of six clinics out of 23 clinics participating in the trial, and the average cost was applied to all 23 clinics to estimate the total CoRECT intervention cost. Thus, potential out‐of‐sample variation in costs is not captured in our cost estimate.

Third, we used microcosting to systematically collect staff time and resources involved in the intervention during all phases of the trial and excluded research costs. Cost collection alongside clinical trials is inherently complex in terms of clearly delineating intervention costs from research costs. The programme cost estimates from microcosting are generally lower than those estimated from gross costing, programme budget or expenditures [26]. The guidelines for cost‐effectiveness in health and medicine recommend using microcosting to accurately identify, measure and value the resources involved in the intervention, when input costs are integral to the cost analysis [23].

Finally, our costs and cost‐effectiveness analyses are based on three CoRECT project sites. All sites are located in the northeast region of the United States, potentially limiting the generalizability of our results to other locations in the United States or in low‐ and middle‐income countries. However, the microcosting approach used in our analysis, including the sequential steps followed in cost data collection, allocation of costs across D2C activities and healthcare agencies, and cost‐effectiveness estimation for intermediate health outcomes, may be replicated elsewhere. Because the D2C intervention is based on reliable medical records and HIV surveillance data to identify the persons who are out of care and to re‐engage them in HIV care, countries with limited capacity to maintain patients’ medical records and/or HIV surveillance data may face additional challenges to implement the intervention.

## 5 CONCLUSIONS

The Cooperative Re‐Engagement Controlled Trial demonstrated that a collaborative D2C model using routine HIV surveillance data and an active re‐engagement intervention was effective in identifying newly out‐of‐care PWH and re‐engaging them in HIV medical care [16]. The CoRECT intervention involved collaborative efforts and costs of both health departments and HIV care providers. The microcosting and cost‐effectiveness analysis showed that the CoRECT intervention costs were comparable with other similar intervention costs reported in the literature, suggesting that the CoRECT intervention may be a cost‐effective approach to improve HIV care continuum outcomes. The analysis may provide health departments, HIV care providers and programme managers with useful information to plan for and implement a collaborative data‐to‐care model for re‐engaging newly out‐of‐care PWH, which help achieve the EHE goals of reducing the number of new HIV infections in the United States.

## COMPETING INTERESTS

The authors declare no competing interests.

## AUTHORS’ CONTRIBUTIONS

All authors have read and approved the final draft. RKS: Conceptualizing the study, designing the cost analysis plan, collecting and analyzing data, writing original draft, and writing and editing final manuscript; RNF: Conceptualizing the study, designing the cost analysis plan, coordinating data collection, reviewing the results, and reviewing and editing the manuscript. LMR, CL, LN, and NC: Conceptualizing the study, collecting and reporting cost data, coordinating with sites for data collection, reviewing the results, and reviewing and editing the manuscript. KAB, HJ, FLA, AD, MV, and PJW: Conceptualizing the study, reviewing the results, and reviewing and editing the manuscript.

## FUNDING

This work was supported by the Centers for Disease Control and Prevention research grant FOA PS14‐001. The three grantees were the Philadelphia Department of Health, the Massachusetts Department of Health and the Connecticut Department of Health.

## DISCLAIMER

The findings and conclusions in this report are those of the authors and do not necessarily represent the views of the Centers for Disease Control and Prevention.

## PREVIOUS PRESENTATION

Presented in part as a poster presentation at CROI: Conference on Retroviruses and Opportunistic Infections, February 12‐16, 2022, Denver, CO.

## Supporting information


**Table S1**. Average monthly start‐up and recurrent costs of the CoRECT intervention in health departments
**Table S2**. Average monthly start‐up and recurrent costs of the CoRECT intervention in HIV care clinics
**Table S3**. Sensitivity of the CoRECT intervention costs and cost‐effectiveness

## Data Availability

Individual participant data will not be shared because of data use agreements between the CDC, the CoRECT clinical sites and the Philadelphia Department of Health, the Connecticut Department of Health and the Massachusetts Department of Health. The study protocol and cost data collection forms are available upon request from the corresponding author.
