# Supplementary material for: Costs and cost‐effectiveness of a collaborative data‐to‐care intervention for HIV treatment and care in the United States
Source: J Int AIDS Soc. 2023 Jan 22;26(1):e26040. doi: 10.1002/jia2.26040 (PMC9867888; doi:10.1002/jia2.26040)
Supplement: Supplementary file 1 — Table S1. Average monthly start‐up and recurrent costs of the CoRECT intervention in health departments Table S2. Average monthly start‐up and recurrent costs of the CoRECT intervention in HIV care clinics Table S3. Sensitivity of the CoRECT intervention costs and cost‐effectiveness [file JIA2-26-e26040-s001.docx]

**Supplementary Tables**

**Costs and cost-effectiveness of a collaborative data-to-care intervention for HIV treatment and care in the United States**

Ram K. Shrestha, PhD^1^, Robyn Neblett Fanfair, MD, MPH^1^, Liisa Randall, PhD^2^, Crystal Lucas, MSW^3^, Lisa Nichols, MPH^4^, Nasima Camp, MPH^1^, Kathleen A. Brady, MD^3^, Heidi Jenkins^5^, Frederick L. Altice, MD, MA^4^, Alfred DeMaria, MD^2^, Merceditas Villanueva, MD^4^, Paul J. Weidle^1^

We analyzed the cost data separately for the health departments and collaborating clinics, then aggregated them to estimate the annual total cost of the CoRECT intervention for each site. The labor costs were estimated by multiplying the number of hours spent on each intervention activity by the hourly wage rate (including fringe benefits) of the staff person who contributed the time. The costs of durable equipment were amortized over the useful duration of the equipment using straight-line depreciation^1^; pre-implementation training for active public health intervention using DIS or field epidemiologists was assumed to last for a year for cost calculation. All other labor and non-labor costs were aggregated as start-up costs ($/month x 3) incurred during the first quarter after project initiation and recurrent costs incurred 6 and 12 months after project initiation. Because the sites reported recurrent cost data either at 6-month or 12-month cycle or the data reported in those two cycles were virtually the same, we combined the data from two cycles to estimate the recurrent costs ($/month x 9).

**Table S1.** Average monthly start-up and recurrent costs of the CoRECT intervention in health departments

|  | Connecticut | | Massachusetts | | Philadelphia | |
| --- | --- | --- | --- | --- | --- | --- |
|  | Start-up ($) | Recurrent ($) | Start-up ($) | Recurrent ($) | Start-up ($) | Recurrent ($) |
| **Variable cost: labor** |  |  |  |  |  |  |
| **Out-of-care list** |  |  |  |  |  |  |
| Generate surveillance line list | 189 | 151 | 680 | 849 | 536 | 210 |
| Match with clinic list | 189 | 151 | 942 | 1,124 | -- | -- |
| Communicate with clinic for data transmission-initial | 189 | 76 | -- | -- | 255 | 131 |
| Health department preliminary investigation | -- | -- | 628 | 628 | 1,582 | 394 |
| Case Conference | 80 | 80 | 565 | 1,248 | 853 | 1,084 |
| Communicate with clinic-data transmission-final | 95 | 76 | -- | -- | 158 | 158 |
| Data entry of final list | 378 | 76 | 565 | 499 | 1,656 | 1,014 |
| **DIS activities** |  |  |  |  |  |  |
| Records review | 484 | 681 | 23 | 1,317 | 433 | 1,485 |
| Outreach to locate and contact Out-of-care patients | 2,459 | 2,990 | 678 | 1,933 | 675 | 2,946 |
| Out-of-care interview and barriers to care survey | 504 | 871 | 163 | 773 | 3,285 | 2,325 |
| Engagement assistance | 1,008 | 1,060 | 176 | 242 | 534 | 2,325 |
| Follow-up with clinics | 403 | 814 | 81 | 242 | 2,579 | 1,395 |
| Follow-up engagement assistance | 685 | 625 | 81 | 121 | 295 | 1,163 |
| Documentation of engagement assistance in the database | 665 | 1,514 | 163 | 1,450 | 3,329 | 3,488 |
| **Variable cost: non-labor** |  |  |  |  |  |  |
| Office supplies | 332 | 452 | 17 | 17 | 106 | 234 |
| **Fixed cost: labor** |  |  |  |  |  |  |
| Pre-implementation training | 355 | -- | 148 | -- | 113 | -- |
| Project-related meetings | 5,136 | 343 | 4,191 | 1,648 | 5,282 | 3,576 |
| Data management | 2,698 | 5,718 | 600 | 150 | 1,202 | 236 |
| Quality assurance | 890 | 890 | 264 | 250 | 1,602 | 672 |
| General administration | 3,813 | 3,813 | 1,120 | 884 | 1,584 | 394 |
| Project supervision | 2,568 | 343 | 1,443 | 1,769 | 4,420 | 5,340 |
| **Fixed cost: non-labor** |  |  |  |  |  |  |
| Electronic equipment | 347 | 347 | 14 | 14 | 27 | 93 |
| Office space and utilities | 1,175 | 1,045 | 752 | 752 | 1,566 | 1,461 |
| **Total cost** | 24,643 | 22,117 | 13,295 | 15,908 | 32,072 | 30,125 |

The cost data were collected at project start-up within 3 months and recurrent phases, within 6 months and 12 months of the project initiation. The same numbers under Start-up and Recurrent cost columns indicate that costs between the two periods remained the same.

-- is not applicable or data not reported. CoRECT, Cooperative Re-engagement Control Trial; DIS, disease intervention specialist.

**Table S2.** Average monthly start-up and recurrent costs of the CoRECT intervention in HIV care clinics

|  | Connecticut (n=23) | | Massachusetts (n=9) | | Philadelphia (n=8) | |
| --- | --- | --- | --- | --- | --- | --- |
|  | Start-up ($) | Recurrent ($) | Start-up ($) | Recurrent ($) | Start-up ($) | Recurrent ($) |
| ***Variable cost*** |  |  |  |  |  |  |
| **Out-of-care patient identification** |  |  |  |  |  |  |
| Generate out-of-care patient list | 2,000 | 1,797 | 3,478 | 3,803 | 144 | 234 |
| Match with clinic list | 2,243 | 2,049 | -- | -- | 2,740 | 1,208 |
| Communicate with health depart for data transmission | 375 | 350 | 210 | 171 | 183 | 89 |
| Case Conference | 1,306 | 239 | 734 | 574 | 2,548 | 961 |
| **Patient re-engagement** |  |  |  |  |  |  |
| Contacting out-of-care patients | 4,268 | 4,678 | 5,636 | 5,622 | 191 | 716 |
| Follow-up with health department | 700 | 359 | 4,588 | 3,131 | 265 | 894 |
| Other: addressing patients’ concerns | -- | -- | 128 | -- | 40 | 26 |
| ***Fixed cost: labor*** |  |  |  |  |  |  |
| Pre-implementation training and travel | 1,101 | -- | 2,674 | -- | 309 | -- |
| Project-related meetings | 2,571 | 506 | 3,812 | 3,469 | 241 | 168 |
| Data management | 3,993 | 3,409 | 1,228 | 2,287 | 44 | 60 |
| Quality assurance | 3,063 | 1,239 | 630 | 196 | 446 | 218 |
| General administration | 571 | 242 | 1,379 | 402 | 84 | 96 |
| Project supervision | 872 | 107 | 1,495 | 1,057 | 181 | 71 |
| Other: care team coordination for transition | -- | -- | 150 | -- | 287 | 258 |
| **Total cost** | 23,060 | 14,975 | 26,141 | 20,712 | 7,703 | 5,000 |

The cost data were collected at project start-up within 3 months and recurrent phases, within 6 months and 12 months of the project initiation. No. of clinics varied in data reporting cycles, hence the average cost per clinic was calculated.

-- is not applicable or data not reported. CoRECT, Cooperative Re-engagement Control Trial.

**Table S3.** Sensitivity of the CoRECT intervention costs and cost-effectiveness

|  | Connecticut | | Massachusetts | | Philadelphia | |
| --- | --- | --- | --- | --- | --- | --- |
|  | Average Cost | ICER | Average Cost | ICER | Average Cost | ICER |
| Base case | 5,765 | 32,669 | 5,634 | 33,807 | 4,482 | 14,169 |
| Reduction in out-of-care patient identification cost |  |  |  |  |  |  |
| Moderate (25% reduction) | 5,569 | 31,560 | 5,327 | 31,959 | 4,275 | 13,513 |
| Low (50% reduction) | 5,374 | 30,452 | 5,019 | 30,111 | 4,067 | 12,858 |
| Reduction in fixed cost |  |  |  |  |  |  |
| Moderate (25% reduction) | 4,993 | 28,293 | 5,036 | 30,218 | 4,049 | 12,800 |
| Low (50% reduction) | 4,221 | 23,916 | 4,438 | 26,628 | 3,616 | 11,431 |
| Contribution of clinic cost |  |  |  |  |  |  |
| Moderate (50% of total) | 6,132 | 34,747 | 5,012 | 30,075 | 6,000 | 18,967 |
| Low (25% of total) | 4,690 | 26,579 | 3,604 | 21,623 | 4,879 | 15,425 |

CoRECT, Cooperative Re-engagement Control Trial; ICER, incremental cost-effectiveness ratio, reported as incremental cost per person re-engaged in HIV care.

**References (Supporting Information):**

1. Walker D, Kumaranayake L. Allowing for differential timing in cost analyses: discounting and annualization. Health Policy Plan. 2002 Mar;17(1):112-8. doi: 10.1093/heapol/17.1.112. PMID: 11861593.
